# Th1 and Th2 Cytokine Profiles in Ewes with Subclinical Mastitis Caused by *Mycoplasma agalactiae* and Coagulase-Negative Staphylococci

**DOI:** 10.3390/vetsci13090942

**Published:** 2026-09-10

**Authors:** Oznur Yilmaz Koc, Tarik Safak, Ahmet Eser, Ozgul Gulaydin, Ali Risvanli

**Affiliations:** 1 Department of Obstetrics and Gynecology, Faculty of Veterinary Medicine, Siirt University, 56100 Siirt, Türkiye; 2 Department of Obstetrics and Gynecology, Faculty of Veterinary Medicine, Kastamonu University, 37150 Kastamonu, Türkiye; tsafak@kastamonu.edu.tr; 3 Department of Reproduction and Artificial Insemination, Faculty of Veterinary Medicine, Siirt University, 56100 Siirt, Türkiye; ahmet.eser@siirt.edu.tr; 4 Department of Microbiology, Faculty of Veterinary Medicine, Siirt University, 56100 Siirt, Türkiye; ozgul.gulaydin@siirt.edu.tr; 5 Department of Obstetrics and Gynecology, Faculty of Veterinary Medicine, Fırat University, 23119 Elazığ, Türkiye; arisvanli@firat.edu.tr

**Keywords:** ewe, cytokine, *Mycoplasma agalactiae*, CoNS

## Abstract

The present study aimed to evaluate Th1- and Th2-associated cytokine profiles in sheep with subclinical mastitis caused by coagulase-negative staphylococci (CoNS) and *Mycoplasma agalactiae* (*M. agalactiae*). The study included 90 Hamdani ewes with subclinical mastitis at mid lactation and without apparent clinical signs. First, the California Mastitis Test (CMT) was performed on each mammary half. Following evaluation of the CMT results, milk samples were collected for microbiological analysis after disinfection of the teat ends. Milk samples that showed no bacterial growth on microbiological analysis and were CMT-negative constituted the control group (n = 30). Milk samples that were CMT-positive and yielded either CoNS (n = 30) or *M. agalactiae* (n = 30) were assigned to the respective study groups. Samples showing growth of two or more bacterial species were excluded from the study. Following completion of sample collection for the predefined groups, Th1-associated cytokines, including Tumor Necrosis Factor-Alpha (TNF-α), Interferon-gamma (IFN-γ), and Interleukine-2 (IL-2), and Th2-associated cytokines, including IL-4, IL-5, and IL-10, were quantified in the milk samples using the enzyme-linked immunosorbent assay (ELISA) method. ELISA analyses were performed in accordance with the manufacturer’s instructions. Comparative analysis of cytokine concentrations among the groups revealed statistically significant differences in IL-2, IL-4, and IL-10 concentrations. Specifically, IL-2, IL-4, and IL-10 concentrations were significantly higher in the CoNS group than in both the control and *M. agalactiae* groups. The CoNS group exhibited significantly higher concentrations of IL-2, IL-4, and IL-10 (12.69 ± 0.50, 66.90 ± 2.43, and 52.45 ± 2.14 ng/L, respectively) compared with the control group (7.50 ± 0.31, 54.62 ± 1.71, and 39.60 ± 2.38 ng/L, respectively) and the *M. agalactiae* group (8.97 ± 0.49, 53.96 ± 2.12, and 42.82 ± 1.83 ng/L, respectively). In contrast, no statistically significant differences were observed among the groups in terms of IFN-γ, TNF-α, and IL-5 concentrations. Overall, these findings suggest that the immune response associated with subclinical mastitis may vary depending on the etiological agent, with distinct cytokine concentration profiles observed between CoNS- and *M. agalactiae*-associated infections.

## Simple Summary

Mastitis is a significant inflammatory condition of the mammary gland that causes damage to milk-producing cells, resulting in alterations in milk composition and a reduction in milk yield. In contrast, subclinical mastitis is characterized by the absence of apparent clinical signs, often making it difficult to detect, while still causing inflammatory damage to mammary tissue and potentially resulting in substantial economic losses. Among the bacterial agents associated with subclinical mastitis, coagulase-negative staphylococci (CoNS) are frequently isolated, whereas *Mycoplasma agalactiae* (*M. agalactiae*) is of particular concern due to its potential to cause substantial damage to mammary tissue and adversely affect milk production, particularly as the infection progresses. This study aimed to evaluate the effects of CoNS- and *M. agalactiae*-associated subclinical mastitis on Th1 and Th2 related immune responses in sheep. For this purpose, milk samples were collected from Hamdani sheep and classified into control, CoNS, and *M. agalactiae* groups based on the presence or absence of bacterial growth and the bacterial agents identified. Once the required sample size was reached in each group, the concentrations of the Th1-related cytokines Interferon Gamma, Tumor Necrosis Factor Alpha, and Interleukine (IL)-2 and the Th2-related cytokines IL-4, IL-5, and IL-10 were determined using enzyme-linked immunosorbent assays. Additionally, the IFN-γ/IL-10 ratio was calculated as an indicator of the balance between proinflammatory and regulatory immune responses. The results demonstrated that cytokine profiles in sheep with subclinical mastitis varied between CoNS- and *M. agalactiae*-associated cases, suggesting that the bacterial agent may influence the local immune response. Evaluation of the IFN-γ/IL-10 ratio provided an additional indicator of the relative predominance of proinflammatory or regulatory immune responses in the two bacterial groups. These findings may contribute to a better understanding of bacteria-specific immune response patterns in subclinical mastitis and provide a basis for further research into more targeted strategies for disease control and modulation of the immune response.

## 1. Introduction

Mastitis is an inflammatory disorder of the mammary gland that can impair the function of milk-producing cells, resulting in alterations in milk production, composition, and quality, and may also cause pathological changes in the udder. Mastitis is a major health problem affecting dairy production worldwide and has significant economic implications for producers due to substantial financial losses associated with reduced milk yield and quality [1,2,3,4]. Mastitis can occur in either clinical or subclinical forms. Clinical mastitis is characterized by varying degrees of visible abnormalities in the udder and milk, which may be accompanied by systemic signs in severe cases. In contrast, subclinical mastitis is characterized by the absence of apparent clinical abnormalities in the udder and milk despite the presence of an intramammary infection and inflammatory response [5,6,7,8].

Subclinical mastitis is characterized by intramammary infection without apparent clinical signs and represents an important economic concern in small ruminant production due to its potential to reduce milk yield and quality. Because affected animals may remain clinically normal, infections can persist undetected, highlighting the importance of appropriate diagnostic methods for early detection and effective disease control [9]. Subclinical mastitis is highly prevalent in small ruminants, with reported herd-level prevalence ranging from 18% to 55.20%. Bacterial pathogens play a key role in the development and progression of mastitis [10,11,12]. The reported prevalence of subclinical mastitis varies considerably among regions and production systems. For example, a study conducted in dairy goats in Indonesia reported a prevalence of 68.51% [13]. Coagulase-negative staphylococci (CoNS) are the most frequently isolated bacterial pathogens associated with subclinical mastitis, accounting for 78% of cases in ewes [14,15]. *Mycoplasma agalactiae* (*M. agalactiae*) is an important causative agent of mastitis in small ruminants and can cause substantial economic losses, particularly through reductions in milk yield. In clinically affected animals, the infection may also be associated with keratoconjunctivitis, polyarthritis, and, less frequently, pneumonia. These clinical manifestations and associated production losses highlight the economic importance of *M. agalactiae* infections in small ruminant production [16].

Cytokines play a critical role in regulating the mammary gland immune response during mastitis. T lymphocytes, particularly CD4+ T cells, contribute to this response through the differentiation of Th0 cells into Th1 and Th2 subsets. Th1-associated cytokines, including IFN-γ, TNF-α, and IL-2, primarily mediate cellular immunity, whereas Th2-associated cytokines, such as IL-4, IL-5, IL-10, and IL-13, are mainly involved in humoral and regulatory immune responses [17,18,19,20]. The IFN-γ/IL-10 ratio is considered an indicator of the balance between pro-inflammatory and anti-inflammatory immune responses. IFN-γ primarily promotes cellular and pro-inflammatory responses, whereas IL-10 exerts regulatory and anti-inflammatory effects. Thus, a higher IFN-γ/IL-10 ratio may indicate a predominantly pro-inflammatory response, while a lower ratio may reflect greater anti-inflammatory or regulatory activity [21].

Previous studies in cattle have shown that pro- and anti-inflammatory cytokines play important roles in regulating the immune response during mammary gland infection and may serve as potential biomarkers for mastitis diagnosis. However, information on their role in ewe mastitis remains limited [22,23]. Numerous studies in cattle [22] and humans [23] have investigated the potential of these molecules as reliable and sensitive indicators for mastitis diagnosis. However, information regarding their role in mastitis in ewes is still quite limited [24]. Therefore, this study aimed to evaluate Th1 and Th2 cytokine profiles in ewes with subclinical mastitis associated with CoNS and *M. agalactiae*.

## 2. Materials and Methods

### 2.1. Establishing Study Groups

The study was conducted on 2–4-year-old Hamdani ewes in mid-lactation, reared on private farms in Siirt Province. Only clinically healthy animals without concurrent diseases were included. Following udder examination and cleaning, the first streams of milk were discarded, and milk from each mammary half was evaluated using the California Mastitis Test (CMT) as described by Safak et al. [3]. Based on gel formation, udder halves were classified as CMT-negative (−) when no reaction was observed or CMT-positive (+) when scored as +, ++, or +++. Based on the CMT results, milk samples were collected from each teat for microbiological and cytokine analyses. For microbiological examination, the teats were disinfected with 70% alcohol, and approximately 3 mL of milk was collected into sterile plastic tubes. An additional 1 mL of milk was collected into Eppendorf tubes for cytokine analysis. Microbiological samples were transported to the Microbiology Laboratory of the Faculty of Veterinary Medicine, Siirt University, within 2 h, whereas cytokine samples were stored at −20 °C until analysis.

**Figure 1:**
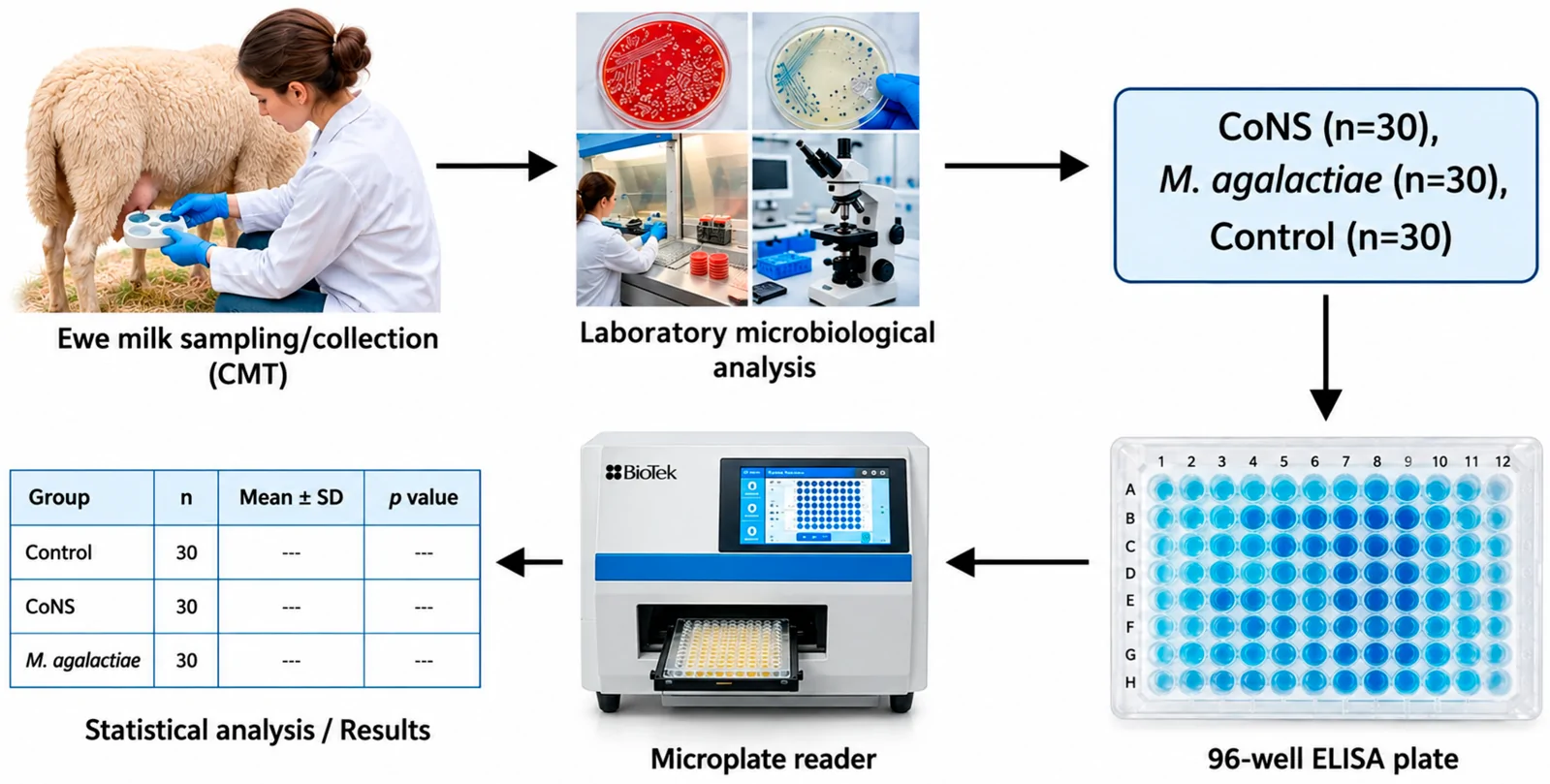
Materials and methods section is presented schematically. CoNS = Coagulase-Negative Stapylococcus, *M. agalactiae* = *Mycoplasma agalactiae*, Th = Helper T.

Group assignment was based on CMT and bacteriological findings. CMT-negative milk samples with no bacterial growth were assigned to the Control group (n = 30), whereas CMT-positive samples yielding only CoNS (n = 30) or *M. agalactiae* (n = 30) were assigned to the respective infected groups. Of the 287 milk samples screened, sampling continued until 30 eligible ewes were obtained for each group, resulting in a final study population of 90 ewes. Only ewes without visible abnormalities in their general condition, udder, or milk were considered to have subclinical mastitis. Ewes with clinical mastitis, characterized by visible abnormalities such as udder swelling, hardness, fever, or changes in milk appearance, were excluded. Animals with other clinically significant diseases, including lameness, pregnancy toxemia, or hypocalcemia, were also excluded. To minimize potential variation related to infection stage, ewes were selected based on positive CMT results and grouped according to their bacteriological findings. An overview of the study design is presented in Figure 1.

### 2.2. Milk Sample Collection, Bacterial Isolation, and Identification

Isolation of *CoNS*: Mastitis-infected milk samples brought to the microbiology laboratory were homogenized using a vortex mixer, then inoculated onto blood agar (Oxoid, CM0271B, Basingstoke, Hampshire, UK) and mannitol salt agar (Oxoid, CM0085B, Basingstoke, Hampshire, UK) containing 5–7% defibrinated ewe blood, and incubated aerobically for 24–48 h. Pure cultures were obtained from agar media in which bacterial growth was observed at the end of the incubation period. Colonies obtained from pure cultures were evaluated according to Gram staining, macroscopic and microscopic morphology, and catalase and coagulase reactions. Accordingly, isolates that were Gram-positive, had cocci morphology, showed a positive catalase reaction, a negative tube coagulase test, and formed pink colonies on mannitol salt agar medium were evaluated as *coagulase-negative Staphylococcus* spp. [25].

Isolation and identification of *M. agalactiae*: Mastitis milk samples were vortexed and inoculated into Mycoplasma Broth Base (Oxoid, CM0403, Basingstoke, Hampshire, UK) supplemented with Mycoplasma Supplement (Oxoid, SR0059C, Basingstoke, Hampshire, UK) and incubated at 37 °C for 7 days under 5–10% CO_2_. The cultures were then inoculated onto Mycoplasma Agar Base (Oxoid, CM0401B, Basingstoke, Hampshire, UK) supplemented with Mycoplasma Supplement and incubated at 37 °C for 7–10 days under 5–10% CO_2_. Following incubation, agar plates were examined by stereomicroscopy at 40× magnification. Colonies exhibiting a characteristic “fried-egg” appearance were considered presumptive *Mycoplasma* spp. [26]. Suspected isolates were identified as *M. agalactiae* using PCR according to Tola et al. [27].

Genomic DNA was extracted from presumptive *M. agalactiae* isolates using a commercial kit (GeneAll, Exgene™ Cell SV 106-152, Seoul, Republic of Korea) according to the manufacturer’s instructions. Identification was performed by PCR targeting the *p80* gene using specific primers [27]. The PCR mixture contained 12.5 µL of 2× PCR Mastermix (ABT^®^, Ankara, Türkiye), 5 µL of genomic DNA, 1.5 µL of each primer (10 µM), and nuclease-free PCR water to a final volume of 25 µL. PCR amplification consisted of an initial denaturation at 94 °C for 10 min, followed by 35 cycles of denaturation at 94 °C for 1 min, annealing at 57 °C for 1 min, and extension at 72 °C for 1 min, with a final extension at 72 °C for 15 min. Nuclease-free water and DNA from *M. agalactiae* ATCC^®^ 35890 were used as negative and positive controls, respectively. PCR products were separated by agarose gel electrophoresis (80 V, 1.5 h) with GelRed^®^ staining and visualized using a gel imaging system (Gen-Box ImagER, Ankara, Türkiye). Isolates yielding the expected 375-bp amplicon were confirmed as *M. agalactiae*.

### 2.3. Cytokine Analyses

The concentrations of Th1-associated cytokines (TNF-α, IFN-γ, and IL-2) and Th2-associated cytokines (IL-4, IL-5, and IL-10) in milk samples were determined using commercially available species-specific ELISA kits (Shanghai YL Biotech Co., Ltd., Shanghai, China) according to the manufacturer’s instructions. Absorbance was measured using an ELISA microplate reader (BioTek Instruments, Winooski, VT, USA), and cytokine concentrations were calculated from the corresponding standard curves.

The inter-assay and intra-assay coefficients of variation were 10% and 8%, respectively. The assay ranges were 5–600 pg/mL for IFN-γ and IL-4, 5–400 ng/L for TNF-α, 2–160 ng/L for IL-2, 1–300 ng/L for IL-5, and 2–700 ng/L for IL-10. ELISA analyses were performed according to the manufacturer’s instructions, and absorbance was measured at 450 nm using an automatic microplate reader (BioTek Instruments, Winooski, VT, USA). All laboratory analyses were conducted at the Laboratory of the Department of Obstetrics and Gynecology, Faculty of Veterinary Medicine, Siirt University [11].

### 2.4. Statistical Analysis

**Figure 2:**
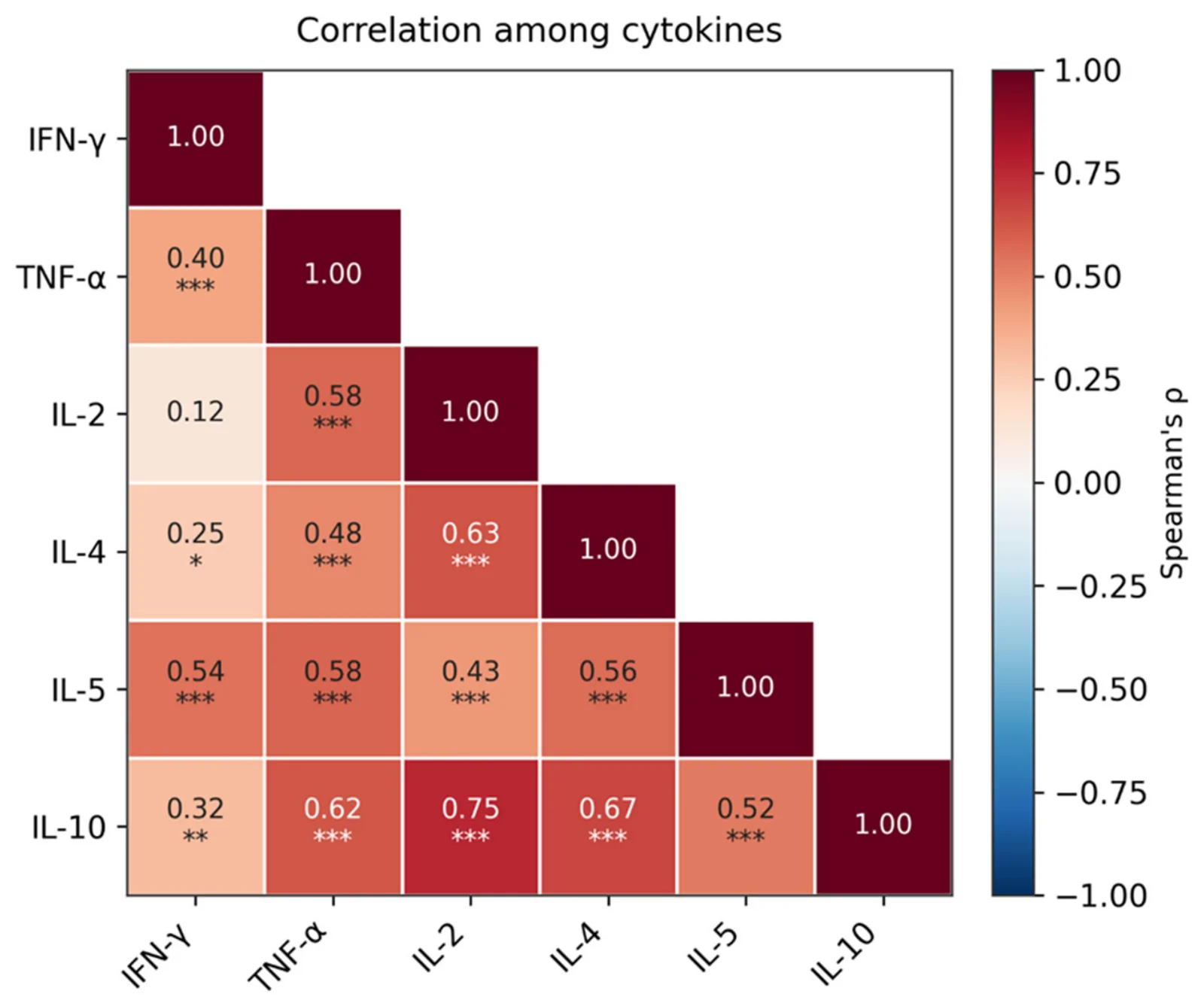
Spearman rank correlation matrix of the six cytokines measured in ewe milk samples (pooled sample, n = 90). Each cell shows the correlation coefficient (ρ) between the corresponding cytokine pair; only the lower triangle is displayed. Color intensity is proportional to the strength and direction of the correlation, as indicated by the scale bar (red, positive; blue, negative). Asterisks denote the level of statistical significance (* *p* < 0.05, ** *p* < 0.01, *** *p* < 0.001). The strongest positive associations were observed among IL-2, IL-4, and IL-10, whereas IFN-γ was weakly correlated with the other cytokines. IFN-γ, interferon-gamma; TNF-α, tumor necrosis factor-alpha; IL, interleukine.

**Figure 3:**
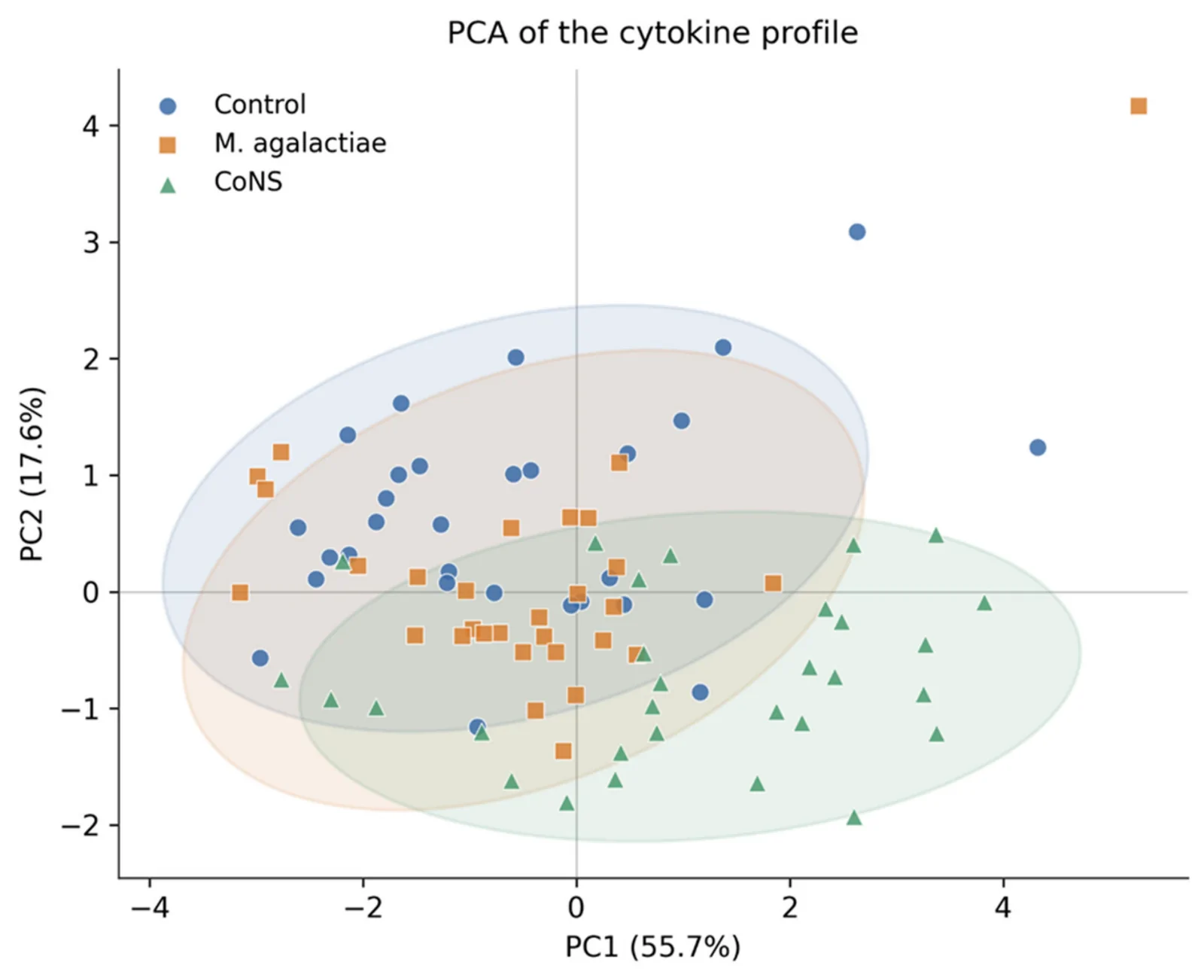
Principal component analysis (PCA) score plot based on the standardized concentrations of the six cytokines in the Control, *Mycoplasma agalactiae* (*M. agalactiae*), and *coagulase-negative staphylococci* (*CoNS*) groups (n = 30 per group). Each symbol represents an individual animal (circles, Control; squares, *M. agalactiae*; triangles, *CoNS*), and shaded ellipses denote the 95% confidence region for each group. The first two principal components together explained 73.4% of the total variance (PC1, 55.7%; PC2, 17.6%). Ewes of the *CoNS* group were separated along PC1 from the Control and *M. agalactiae* groups, which overlapped substantially, reflecting the distinct cytokine profile of the *CoNS* group.

Data are presented as mean ± SEM. Normality (Shapiro–Wilk) and homogeneity of variance (Levene) were assessed for each cytokine (Supplementary Table S1). As variances were homogeneous, group differences were evaluated by one-way ANOVA with Tukey’s HSD post-hoc test; *p*-values were adjusted across the six cytokines using the Benjamini–Hochberg false discovery rate (FDR) procedure, and eta-squared (η^2^) was reported as the effect size for all cytokines (Table 1). The presence of an overall group difference was interpreted from the FDR-adjusted omnibus *p*-value, and post-hoc groupings were evaluated only for cytokines that remained significant after correction. Because normality was violated in some groups, the non-parametric Kruskal–Wallis test with Dunn’s post-hoc comparison was additionally performed as a confirmatory analysis (Table 2), which yielded concordant results. The overall effect of group on the cytokine profile was examined by MANOVA, inter-cytokine relationships by Spearman’s rank correlation (Figure 2), and the multivariate structure by principal component analysis (Figure 3). Analyses were performed in SPSS Statistics v. 22 (IBM Corp., Armonk, NY, USA); *p* < 0.05 was considered statistically significant.

## 3. Results

MANOVA revealed a highly significant overall effect of infection status on the combined six-cytokine profile (Wilks’ λ = 0.348, F_12_,_164_ = 9.49, *p* < 0.001), justifying subsequent univariate comparisons. Group-wise concentrations, post-hoc groupings, and effect sizes are summarized in Table 1, and all pairwise comparisons with their standardized effect sizes are presented in Table 2.

Three cytokines differed significantly among groups after false discovery rate (FDR) correction, and all showed the same pattern of elevation in the *CoNS* group (Table 1). IL-2 concentrations were markedly higher in *CoNS* ewes (12.69 ± 0.50 ng/L) than in the Control (7.50 ± 0.31 ng/L) and *M. agalactiae* (8.97 ± 0.49 ng/L) groups (both *p* < 0.001; Hedges’ g = −2.25 and −1.35, respectively; Table 2), whereas the Control and *M. agalactiae* groups did not differ from each other (*p* = 0.055). A comparable pattern was observed for IL-4, which was significantly elevated in *CoNS* ewes (66.90 ± 2.43 ng/L) relative to both the Control (54.62 ± 1.71 ng/L) and *M. agalactiae* (53.96 ± 2.12 ng/L) groups (both *p* < 0.001; Hedges’ g = −1.05 and −1.02, respectively; Table 2). Likewise, IL-10 was highest in the *CoNS* group (52.45 ± 2.14 ng/L) compared with the Control (39.60 ± 2.38 ng/L; *p* < 0.001; g = −1.02) and *M. agalactiae* (42.82 ± 1.83 ng/L; *p* = 0.005; g = −0.87) groups (Table 2).

In contrast, IFN-γ, TNF-α, and IL-5 did not differ significantly among the groups after FDR correction of the omnibus ANOVA (adjusted *p* = 0.170, 0.090, and 0.430, respectively), with correspondingly small effect sizes (η^2^ ≤ 0.06; Table 1). For TNF-α, a marginal *M. agalactiae* vs. CoNS post-hoc difference was observed (Tukey *p* = 0.048, not adjusted across cytokines; Table 2); however, because the TNF-α omnibus test did not remain significant after FDR correction, this comparison was regarded as exploratory and was not interpreted as a confirmed group difference. For the three cytokines that were significant in the omnibus analysis (IL-2, IL-4, and IL-10), the parametric (Tukey HSD) and non-parametric (Dunn) post-hoc tests were concordant across all pairwise comparisons, confirming the robustness of these findings (Table 2).

Spearman correlation analysis indicated that the cytokines were broadly positively interrelated (Figure 2). The strongest associations were observed among IL-2, IL-4, and IL-10 (IL-2–IL-10, ρ = 0.75; IL-4–IL-10, ρ = 0.67; IL-2–IL-4, ρ = 0.63; all *p* < 0.001), the same three mediators that discriminated the CoNS group, suggesting a coordinately regulated response. In contrast, IFN-γ showed the weakest associations with the other cytokines (e.g., IFN-γ–IL-2, ρ = 0.12, *p* > 0.05; Figure 2).

Principal component analysis of the six-cytokine profile captured 73.4% of the total variance in the first two components (PC1, 55.7%; PC2, 17.6%). As shown in Figure 3, ewes of the *CoNS* group were largely displaced toward positive PC1 values and separated from the Control and *M. agalactiae* groups, which overlapped substantially, consistent with the univariate findings.

**Figure 4:**
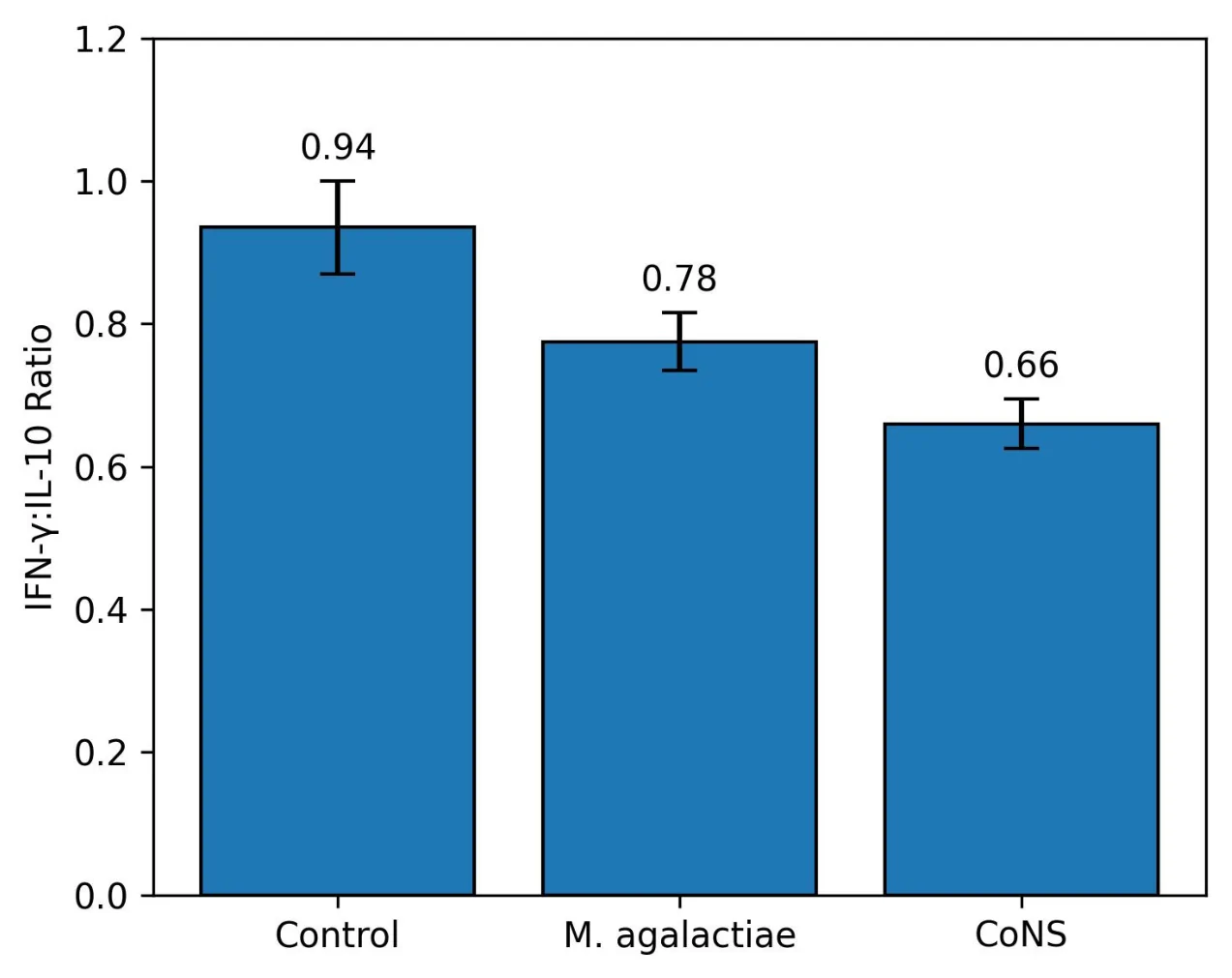
CoNS = Coagulase-Negative Stapylococcus, *M. agalactiae* = *Mycoplasma agalactiae* and control group Th1/Th2 cytokine ratio between groups, which was calculated by dividing Th1 values by Th2 values.

The IFN-γ/IL-10 ratio showed a numerical decrease from the control group (0.94) to the *M. agalactiae* (0.78) and CoNS (0.66) groups. This pattern indicated a relative shift toward an immunoregulatory profile in the infected groups, with the lowest ratio observed in the CoNS group (Figure 4).

## 4. Discussion

Cytokines play an important role in mammary gland immune defense by promoting leukocyte recruitment and enhancing phagocytic and bactericidal activity. Because the mammary immune response varies according to the etiological agent, understanding host–pathogen interactions and local defense mechanisms is important for the diagnosis and control of mammary gland inflammation [28].

Mammary gland defense involves both innate and adaptive immune mechanisms. The innate immune system provides the first line of defense against invading pathogens and acts before the development of an adaptive immune response. The adaptive immune system provides a more specific and effective response upon repeated exposure to previously encountered pathogens. Although innate and adaptive immunity have distinct functions, they are closely interconnected and work together through shared pathways and mechanisms. Mammary gland defense involves a range of innate and adaptive mechanisms, including both humoral and cellular immune responses [29].

This study evaluated Th1-associated cytokines (IFN-γ, TNF-α, and IL-2), which are primarily involved in cell-mediated immunity, and Th2-associated cytokines (IL-4, IL-5, and IL-10), which contribute to humoral and regulatory immune responses. Tumor necrosis factor-α (TNF-α) is a pro-inflammatory cytokine produced primarily by macrophages. It contributes to the regulation of inflammatory responses by promoting the production of other cytokines and modulating cellular functions and tissue homeostasis. Tumor necrosis factor-α (TNF-α) is an important pro-inflammatory cytokine involved in the early immune response to infection. Previous studies have reported significantly higher TNF-α concentrations in cows with clinical and subclinical mastitis compared with healthy animals [30,31]. In ewes, Albenzio et al. [28] reported higher TNF-α concentrations in animals from which bacteria were isolated. Similarly, Alluwaimi et al. [20] showed that TNF-α concentrations increased during the early stages of mastitis and subsequently declined over time, suggesting a time-dependent cytokine response to infection. In our study, the highest CoNS prevalence was observed in the control group, although no significant difference was found between the groups. This finding may be related to the transient nature of pro-inflammatory cytokine responses, which may peak within the first 1–12 h of infection and subsequently decline.

Interferon-γ is a cytokine produced by innate and adaptive immune cells that plays an important role in protective immunity and pathogen elimination [30]. Increased IFN-γ concentrations have been reported in naturally occurring and experimentally induced mastitis caused by various bacterial pathogens, including *Escherichia coli*, *Staphylococcus aureus*, *Mycoplasma bovis*, and *Streptococcus uberis* [22,32,33,34,35]. Franzoni et al. [24] reported that, among ewes categorized according to somatic cell count (SCC), animals with the highest SCC also had the highest IFN-γ concentrations. In contrast, Vitenberga-Verza et al. [31] reported that IFN-γ concentrations were highest in healthy animals, followed by those with subclinical mastitis, and lowest in animals with clinical mastitis. Similarly, Faaz et al. [32] reported significantly higher IFN-γ concentrations in healthy animals than in those with clinical mastitis. Although no statistically significant difference was observed among the groups, IFN-γ concentrations were numerically higher in healthy animals than in the other two groups.

Regarding IL-2, Alluwaimi et al. [20] reported lower concentrations in mastitis cases. In contrast, other studies have reported increased IL-2 concentrations in mastitis caused by *Staphylococcus aureus* and *Escherichia coli*. In our study, IL-2 concentrations differed significantly among all three groups, with the lowest concentration in the control group and the highest in the CoNS group.

Interleukine-4 (IL-4) is an important cytokine involved in humoral immunity, primarily regulating IgE-mediated immune responses and inhibiting IFN-γ production [32]. Studies in cows with mastitis have reported lower IL-4 concentrations in affected animals than in healthy cows [32,36]. Fonseca et al. [37] found no statistically significant difference in IL-4 concentrations between cows with mastitis and healthy cows, although concentrations were numerically lower in mastitic animals. In contrast, Vitenberga-Verza et al. [30] and Şafak and Rişvanlı [22] reported higher IL-4 concentrations in cows with mastitis. Similarly, a study in Barki ewes reported increased IL-4 expression in animals with mastitis [38]. Our study also revealed significant differences in IL-4 concentrations between groups, with significantly higher concentrations in the CoNS group than in the control group. Furthermore, the IFN-γ/IL-4 balance observed in our study was consistent with these findings.

Interleukine-10 is an important anti-inflammatory cytokine involved in the regulation of immune responses and the Th1/Th2 balance [36]. It suppresses Th1-associated cytokines, including IFN-γ and IL-12, thereby contributing to the regulation of inflammatory responses and promoting a shift toward a Th2-dominant response [22,30,39]. Some studies have reported lower IL-10 concentrations in mastitic animals than in healthy animals [31]. In contrast, other studies have reported higher IL-10 concentrations in mastitic animals than in healthy animals [22,32,37,39,40]. However, Bochniarz et al. [36] reported lower IL-10 concentrations in milk from ewes with CoNS mastitis compared with healthy animals, which differs from our findings. These changes in IL-10 concentrations may be associated with its increased anti-inflammatory activity during the later stages of infection. In our study, IL-10 concentrations differed significantly among the groups, with the highest concentration observed in the CoNS group and lower concentrations in the control and *M. agalactiae* groups. Higher IL-10 levels during mid-lactation may reflect its role in regulating mammary inflammation and maintaining immune homeostasis, as supported by Albenzio et al. [28]. Monitoring milk cytokine profiles may provide insights into the local immune status and inflammatory responses of the mammary gland. The significantly higher IL-10 concentration observed in the CoNS group, together with elevated pro-inflammatory cytokine concentrations, may reflect a concomitant regulatory response to inflammation. This increase in IL-10 may represent a compensatory mechanism that limits excessive inflammatory activity and helps maintain immune homeostasis in the mammary gland. Furthermore, the relative relationship between IL-10 and IFN-γ may provide insight into cytokine polarization and the Th1/Th2 balance [21].

This study has some limitations related to the multifactorial nature of mastitis. Species-level identification of CoNS isolates was not performed, as the bacteriological analysis focused on identifying the bacterial group rather than determining individual CoNS species. Therefore, potential species-specific differences in cytokine responses could not be evaluated. Therefore, classification as CoNS was considered sufficient for group assignment. Nevertheless, the lack of species-level identification limits the evaluation of potential species-specific differences in pathogenicity and immune responses. Moreover, cytokine responses may be influenced by several factors, including pathogen characteristics, host genetic and physiological status, and the duration and severity of infection. In the present study, longitudinal monitoring was not performed, and the duration or stage of infection was not determined. Therefore, the temporal dynamics of cytokine responses and their relationship with disease progression could not be assessed. Accordingly, the cytokine profiles observed should be interpreted as reflecting the immune status of the animals at the time of sampling rather than changes occurring throughout the course of infection. A further limitation concerns the sample size: although n = 30 per group provided adequate statistical power for the moderate-to-large effects observed (achieved power > 0.98 for IL-2, IL-4, and IL-10), the study was not powered to detect small differences, and the non-significant findings for IFN-γ, TNF-α, and IL-5 (η^2^ ≤ 0.06) should therefore be interpreted with caution rather than as evidence of no difference. In addition, only a limited panel of Th1- and Th2-associated cytokines was evaluated, and more comprehensive analyses were not feasible because of cost and technical constraints. Furthermore, the limited literature on cytokine responses associated with *M. agalactiae*-induced mastitis restricted direct comparison of our findings with previous studies. Importantly, milk cytokine concentrations reflect the local cytokine milieu and should not be interpreted as direct evidence of cellular Th1/Th2 polarization. Therefore, the cytokine patterns observed in this study, including changes in the IFN-γ/IL-10 ratio, should be interpreted as indicators of relative inflammatory and immunoregulatory activity rather than definitive evidence of Th1/Th2 polarization. Future studies should validate these findings using larger sample sizes and by integrating clinical and epidemiological data, particularly SCC. Future longitudinal studies should determine the duration and stage of infection, characterize CoNS isolates at the species level, and assess factors such as age, breed, parity, lactation stage, and season to better understand their potential influence on cytokine responses. Furthermore, longitudinal assessment of cytokine kinetics in milk from ewes with subclinical mastitis would help elucidate the temporal dynamics of the local immune response.

## 5. Conclusions

In conclusion, this study demonstrated that milk cytokine profiles in ewes with subclinical mastitis associated with *M. agalactiae* and CoNS varied according to the bacterial agent. The observed differences may be related to variation in the stage of infection, particularly whether *M. agalactiae*-infected ewes were in the early or later stages of infection. Furthermore, the progressive decrease in the IFN-γ/IL-10 ratio from the control group to the *M. agalactiae* and CoNS groups indicates a relative shift toward an immunoregulatory/anti-inflammatory cytokine profile in infected animals. The markedly lower ratio in the CoNS group suggests a greater relative contribution of IL-10-mediated immunoregulatory activity to the local cytokine response. In the present study, the higher concentrations of both Th1-associated (IL-2) and Th2-associated (IL-4 and IL-10) cytokines in the CoNS group may reflect a concomitant activation of pro-inflammatory and immunoregulatory responses rather than a clearly Th1- or Th2-dominant response. Mastitis remains a major challenge in dairy production; therefore, further studies are warranted to better characterize cytokine responses associated with different bacterial pathogens in ewe mastitis. The findings of the present study may provide a basis for future research on pathogen-specific immune responses in subclinical mastitis.

## Figures and Tables

**Table 1:** Milk cytokine concentrations (mean ± SEM) in ewes of the Control, *Mycoplasma agalactiae*, and coagulase-negative staphylococci (CoNS) groups (n = 30 per group).

Cytokine	Control	*M. agalactiae*	CoNS	*p*	*η* ^2^
IFN-γ	37.00 ± 1.33	33.51 ± 1.25	34.64 ± 1.18	0.170	0.04
TNF-α	27.14 ± 2.29	23.40 ± 1.34	29.91 ± 1.99	0.090	0.06
IL-2	7.50 ± 0.31 ^a^	8.97 ± 0.49 ^a^	12.69 ± 0.50 ^b^	**<0.001**	0.46
IL-4	54.62 ± 1.71 ^a^	53.96 ± 2.12 ^a^	66.90 ± 2.43 ^b^	**<0.001**	0.22
IL-5	30.66 ± 1.30	32.51 ± 1.34	32.97 ± 1.33	0.430	0.02
IL-10	39.60 ± 2.38 ^a^	42.82 ± 1.83 ^a^	52.45 ± 2.14 ^b^	**<0.001**	0.18

Values are mean ± SEM (ng/L for IFN-γ, TNF-α, IL-5, IL-2, IL-4, and IL-10). Superscript letters are shown only for cytokines with a significant omnibus effect after FDR correction; within such a row, means not sharing a common letter differ significantly (Tukey HSD, *p* < 0.05). *p*, one-way ANOVA with Benjamini–Hochberg FDR correction across the six cytokines; η^2^, eta-squared effect size. Exploratory pairwise differences for cytokines with a non-significant omnibus test (e.g., TNF-α) are reported only in Table 2.

**Table 2:** Pairwise post-hoc comparisons of cytokine concentrations among groups: Tukey HSD (primary; Hedges’ g) and Dunn’s test (confirmatory; Cliff’s δ).

Cytokine	Comparison	*p* (Tukey)	*Hedges’ g*	*p* (Dunn)	*Cliff’s δ*
IFN-γ	Control vs. *M. agalactiae*	0.128	0.49	0.045	0.37
	Control vs. CoNS	0.384	0.34	0.827	0.16
	*M. agalactiae* vs. CoNS	0.803	−0.17	0.539	−0.20
TNF-α	Control vs. *M. agalactiae*	0.356	0.36	1.000	0.13
	Control vs. CoNS	0.565	−0.23	0.235	−0.25
	*M. agalactiae* vs. CoNS	**0.048**	−0.69	0.019	−0.42
IL-2	Control vs. *M. agalactiae*	0.055	−0.64	0.118	−0.38
	Control vs. CoNS	**<0.001**	−2.25	<0.001	−0.91
	*M. agalactiae* vs. CoNS	**<0.001**	−1.35	<0.001	−0.74
IL-4	Control vs. *M. agalactiae*	0.973	0.06	1.000	−0.04
	Control vs. CoNS	**<0.001**	−1.05	<0.001	−0.54
	*M. agalactiae* vs. CoNS	**<0.001**	−1.02	<0.001	−0.59
IL-5	Control vs. *M. agalactiae*	0.586	−0.25	1.000	−0.15
	Control vs. CoNS	0.437	−0.32	0.223	−0.24
	*M. agalactiae* vs. CoNS	0.968	−0.06	1.000	−0.17
IL-10	Control vs. *M. agalactiae*	0.535	−0.27	0.384	−0.27
	Control vs. CoNS	**<0.001**	−1.02	<0.001	−0.64
	*M. agalactiae* vs. CoNS	**0.005**	−0.87	0.008	−0.49

Tukey HSD *p*-values accompany the primary one-way ANOVA; Dunn’s *p*-values (Bonferroni-adjusted) accompany the confirmatory Kruskal–Wallis analysis. Tukey HSD *p*-values are adjusted for the three pairwise comparisons within each cytokine but not across the six-cytokine family; the interpretation of overall group differences was based on the FDR-adjusted omnibus *p*-values reported in Table 1. Accordingly, the marginal *M. agalactiae* vs. CoNS difference for TNF-α (Tukey *p* = 0.048) is regarded as exploratory, because the TNF-α omnibus test did not remain significant after FDR correction (Table 1). Hedges’ g (bias-corrected standardized mean difference): |g| ≈ 0.2 small, 0.5 medium, 0.8 large. Cliff’s δ (non-parametric effect size): |δ| ≈ 0.15 small, 0.33 medium, 0.47 large. Bold denotes Tukey *p* < 0.05.

## Data Availability

The original contributions presented in this study are included in the article. Further inquiries can be directed to the corresponding author.

## Supplementary Materials

The following supporting information can be downloaded at: https://www.mdpi.com/article/10.3390/vetsci13090942/s1. Table S1: Assessment of ANOVA assumptions for each cytokine: Shapiro–Wilk test of normality (per group) and Levene’s test of homogeneity of variance across groups.

## Abbreviations

Th	T Helper
CoNS	Coagulase-Negative Staphylococci
*M. agalactiae*	*Mycoplasma agalactiae*
CMT	California Mastitis Test (CMT)
TNF-α	Tumor Necrosis Factor Alpha
IL	Interleukine
IFN-γ	Interferon gamma
FDR	False Discovery Rate
ANOVA	Analysis of Variance
MANOVA	Multivariate Analysis of Variance
PCA	Principal component analysis
SCC	Somatic Cell Count
