# Supplementary material for: Th1 and Th2 Cytokine Profiles in Ewes with Subclinical Mastitis Caused by Mycoplasma agalactiae and Coagulase-Negative Staphylococci
Source: Vet Sci. 2026 Sep 10;13(9):942. doi: 10.3390/vetsci13090942 (PMC13612004; doi:10.3390/vetsci13090942)
Supplement: Supplementary file 1 [file vetsci-13-00942-s001.zip › vetsci-4509807-supplementary.pdf]

Supplementary Table S1. Assessment of ANOVA assumptions for each cytokine: Shapiro–Wilk test of normality (per group) and Levene's test of homogeneity of variance across groups.

**Cytokine**	**Control**	***M. agalactiae***	**CoNS**	**Levene**
	W (p)	*W (p)*	*W (p)*	*W (p)*
IFN-γ	0.978 (0.774)	0.650 (**<0.001**)	0.967 (0.461)	1.256 (0.290)
TNF-α	0.734 (**<0.001**)	0.857 (**<0.001**)	0.859 (**<0.001**)	0.962 (0.386)
IL-2	0.987 (0.968)	0.849 (**<0.001**)	0.966 (0.431)	1.588 (0.210)
IL-4	0.906 (**0.012**)	0.912 (**0.017**)	0.924 (**0.035**)	0.506 (0.605)
IL-5	0.940 (0.091)	0.702 (**<0.001**)	0.973 (0.626)	0.898 (0.411)
IL-10	0.793 (**<0.001**)	0.941 (0.099)	0.969 (0.508)	0.245 (0.783)

Note. Values are the Shapiro–Wilk W statistic with the corresponding p-value in parentheses (Levene's test is median-centred, reported as the W statistic with its p-value). Bold p-values indicate a significant departure from normality ($p < 0.05$). Normality was rejected in at least one group for all six cytokines, whereas homogeneity of variance was satisfied for every cytokine (all Levene $p > 0.05$). This pattern (variance homogeneity met but normality not met) is the basis for using one-way ANOVA as the primary test while additionally reporting the non-parametric Kruskal–Wallis test as a confirmatory (sensitivity) analysis. $n = 30$ per group.
